# Bacterial Outer Membrane Vesicles and Vaccine Applications

**DOI:** 10.3389/fimmu.2014.00121

**Published:** 2014-03-24

**Authors:** Reinaldo Acevedo, Sonsire Fernández, Caridad Zayas, Armando Acosta, Maria Elena Sarmiento, Valerie A. Ferro, Einar Rosenqvist, Concepcion Campa, Daniel Cardoso, Luis Garcia, Jose Luis Perez

**Affiliations:** ^1^Finlay Institute, Havana, Cuba; ^2^Strathclyde Institute of Pharmacy and Biomedical Science, Strathclyde University, Glasgow, UK; ^3^Norwegian Institute of Public Health, Oslo, Norway

**Keywords:** outer membrane vesicles, vaccines, proteoliposomes, adjuvant, *Neisseria meningitidis*, *Vibrio cholerae*, *Bordetella pertussis*, *Mycobacterium tuberculosis*

## Abstract

Vaccines based on outer membrane vesicles (OMV) were developed more than 20 years ago against *Neisseria meningitidis* serogroup B. These nano-sized structures exhibit remarkable potential for immunomodulation of immune responses and delivery of meningococcal antigens or unrelated antigens incorporated into the vesicle structure. This paper reviews different applications in OMV Research and Development (R&D) and provides examples of OMV developed and evaluated at the Finlay Institute in Cuba. A Good Manufacturing Practice (GMP) process was developed at the Finlay Institute to produce OMV from *N. meningitidis* serogroup B (dOMV_B_) using detergent extraction. Subsequently, OMV from *N. meningitidis*, serogroup A (dOMV_A_), serogroup W (dOMV_W_), and serogroup X (dOMV_X_) were obtained using this process. More recently, the extraction process has also been applied effectively for obtaining OMV on a research scale from *Vibrio cholerae* (dOMV_C_), *Bordetella pertussis* (dOMV_BP_), *Mycobacterium smegmatis* (dOMV_SM_), and BCG (dOMV_BCG_). The immunogenicity of the OMV has been evaluated for specific antibody induction, and together with functional bactericidal and challenge assays in mice has shown their protective potential. dOMV_B_ has been evaluated with non-neisserial antigens, including with a herpes virus type 2 glycoprotein, ovalbumin, and allergens. In conclusion, OMV are proving to be more versatile than first conceived and remain an important technology for development of vaccine candidates.

## Introduction

Vesicles derived from pathogens have been used for a long time in the development of immunogenic vaccine candidates against the respective organisms from which the vesicles have been obtained. Proteoliposomes, outer membrane vesicles (OMV), proteasomes (1), and very small size proteoliposomes (2) are examples of the different approaches of vesicle formulations obtained from microorganisms. Currently, licensed vaccines based on OMV use detergent extraction to obtain dOMV from Gram negative bacteria (3). In addition, it is possible to obtain OMV by inducing the release of “blebs” or native OMV (nOMV) from bacteria (3). A drawback of this latter method is that the resulting vesicles contain a high amount of lipopolysaccharide (LPS), which is very toxic (4). Therefore, several strategies are under evaluation to produce nOMV from mutant strains containing detoxified LPS (5).

Various infectious diseases [such as tuberculosis (TB) and meningitis] and including enteric diseases (such as cholera, salmonellosis, and shigellosis) remain a health problem in children and young adults (6). No vaccines have been developed against the responsible pathogens and the OMV strategy represents a feasible opportunity to address this. OMV are at the interface between traditional and new methods of vaccine production. Antigens and immune stimulator molecules from OMV are extracted from the pathogen and purified in proteolipidic vesicles, a reason for also calling OMV proteoliposomes. Another approach is to use purified molecules from bacteria and inserting them into lipidic nanovesicles or adding any other components to the formulation. Therefore, several research groups have developed structures like proteasomes, which combine *Neisseria meningitidis* protein aggregates with LPS from *Shigella flexneri* (1) or very small size proteoliposomes, which combine OMV, also from *N. meningitidis*, with the ganglioside GM3 more frequently associated with tumor cells (2). The main goal of these nanoparticles or vesicles is to present or deliver their load to competent cells of the immune system (7). This mini-review examines the main developments in various OMV technologies.

## OMV Vaccines Against *Neisseria meningitidis*

Meningococcal disease can occur rapidly following even mild symptoms and can result in fatality and disability. Thus, vaccination is seen as an essential strategy to prevent the rapid onset of infection. Current vaccines against *N. meningitidis* have been developed using the capsular polysaccharide of the pathogen and have been in use since the 1960s against serogroup A and C and since the 1980s against serogroups A, C, Y, and W (8). These structures are highly immunogenic and can be conjugated to carrier proteins to induce memory immune responses and immunogenicity in children younger than 2 years of age (9). However, polysaccharides from the *N. meningitidis* serogroup B (MenB) are low in immunogenicity and safety concerns have arisen due to potential risks of autoimmunity (3). Therefore, novel strategies, based on protein vaccines, have been developed to overcome this hurdle. The use of wild type OMV vaccines against MenB has been explored since the 1970s and public health interventions in countries such as Cuba, Norway, and New Zealand have proven the concept of their efficacy, with high effectiveness estimated in young and adults in the region where the circulating strain was the same as the vaccine strain (8). The Cuban VA-MENGOC-BC^®^ showed 83% effectiveness (over 16 months) in young and adults (10), the Norwegian MenBVac^®^ showed 87% effectiveness (over 10 months) in young and adults (11), and MeNZB administered in New Zealand, showed 73% effectiveness in the young and adults (12). In general, estimates of vaccine efficacy in children and infants are over 70%, although the number of doses required may differ between each vaccine in order to keep protective immunity for a longer period of time, e.g., the Cuban vaccine is administered in two doses, whereas MenBVac^®^ is given in three doses and MeNZB^®^ in four doses (12). These vaccines are examples of parenteral licensed vaccines against meningococcal B disease (details summarized in Table 1).

**Table 1:** OMV vaccines from *N. meningitidis* serogroup B*.

Vaccine name	Developmental history	Comments	Reference
VA-MENGOC-BC^®^	Developed at the Finlay Institute, Cuba, to address an epidemic and tested between 1987 and 1989. The strain type B:4:P1.19,15 was used	Applied in the National Immunization Program of Cuba for more than 20 years	(3, 10)
MenBvac^®^	Developed at the Norwegian Institute of Public Health (NIPH) to address an epidemic and tested between 1988 and 1991. The production strain was the 44/76-SL, type B:15:P1.7,16	Applied in a region of Normandy, France. This technology was used to enable development of MeNZB^®^ and Bexsero^®^ vaccines	(3, 11)
MeNZB^®^	Developed against strain NZ 98/254 (strain type B:4:P1.7-2) and used between 2004 and 2008. The project was a partnership between the WHO, the New Zealand government, the University of Auckland, NIPH, and Chiron	Applied during epidemics in New Zealand. Significant partnership development enabled a high number of clinical trials to be carried out	(8, 12)
Bexsero^®^	Developed by Novartis, and designed to provide broad-based protection. Recently licensed by the European Medicines Agency (www.ema.europa.eu)	Combination of dOMV from strain NZ 98/254 with three recombinant antigens, two of which are fusion proteins (targeting five meningococcal proteins, total: the factor H-binding protein, neisserial adhesin A, and neisserial heparin-binding antigen)	(13)

*The only OMV vaccines licensed to date.

The examples given above are wild type OMV vaccines, obtained using deoxycholate detergent extraction of the bacterial membranes. This method detoxifies and reduces the LPS content in vesicles to amounts proven to be safe by several millions of doses of OMV vaccines administered to humans (10–12, 14). All theses OMV vaccines have been demonstrated to be effective against the epidemic strain, although little or no effect has been found in infants when measuring effectiveness against heterologous strains by SBA (3), thus questioning their broad applicability against a range of circulating MenB strains. The immunodominant antigens in *N. meningitidis* OMV are porins PorA and PorB (14); there is a high variability between these proteins in strains of the same serogroup, therefore, the immune response to OMV is strain specific and some authors have proposed the concept of developing “tailor-made” vaccines against the circulating strain (3, 12). On the other hand, minor proteins in OMV, non-porins, are also responsible for the cross-protection level found in different clinical trials (3) and different strategies, such as the recent Novartis (Switzerland) Bexsero^®^ vaccine (13) uses these minor proteins to construct a more universal vaccine. Several proteomic techniques have been developed to characterize protein antigens to aid the selection of appropriate strains and antigens to improve the extraction protocol (15). It is also known that detergent protocols may not be effective in extracting some important protein antigens such as Factor H binding protein (Fhbp), whereas other protocols (no detergents) permit extraction and inclusion of this antigen in the vesicles. Overall, it is very beneficial to remove endotoxins and allow inclusion of immunogenic antigens in OMV. The advantages of free detergent technologies or inclusion of recombinant proteins to dOMV are under evaluation in new candidates and licensed vaccines, respectively (13, 16).

The Bexsero^®^ vaccine combines OMV that have been classically extracted by detergent and inclusion of recombinant antigens designed by reverse vaccinology (13). The recombinant antigens induce immune responses to a high number of serogroup B strains and the OMV potentiate the immune response to them. Novel strategies are envisaged to obtain OMV from recombinant *N. meningitidis* strains, where LPS has been genetically detoxified (lpx1-mutants), avoiding the need for detergent extraction. Furthermore, mutant strains with over-expressed protein vaccine antigens, like PorA and Fhbp, naturally inserted into the membranes have been constructed (16). Certainly, a high number of vaccine candidates with these characteristics will be seen in the next few years.

More recently, the Finlay Institute (Cuba) and the Norwegian Institute of Public Health (NIPH, Norway) have been working together to develop multivalent OMV vaccines against serogroups A (dOMV_A_), W (dOMV_W_), and X (dOMV_X_) (17, 18). These serogroups represent the main cause of meningococcal disease in Africa (19). A Phase I clinical trial commenced at the end of 2013 to evaluate the safety of a bivalent candidate against serogroups A and W and the results are currently being examined (20). These dOMV were obtained using epidemic strains isolated in countries from the African “meningitis belt”: dOMV_A_ were developed from strain MK499/03, sequence type (ST) 5 clonal complex (cc) and dOMV_W_ were developed from strain MK222/02, ST11 cc (21). All the strains belong to clonal complexes of serogroups that caused epidemics and outbreaks several years ago (22). On the other hand, new cases of meningococcal disease are produced by serogroup X in countries from the African meningitis belt (23). Based on previous experience with a combination of dOMV_A_ and dOMV_W_, both teams have begun research into a new combination including dOMV from meningococcal serogroup X strain BF 2/97 (cc.181) (17).

## Other Applications

Since MenB OMV have had significant exposure to humans in clinical trials, it is reasonable to assume that the safety and tolerability profile would encourage development of other applications. Thus, taking advantage of the immune stimulating molecules present in OMV. Since few adjuvants are licensed for human use, it was a reasonable concept to examine the potential of OMV for adjuvant activity. The adjuvant potential of MenB OMV (OMV_B_) have therefore been demonstrated with non-neisserial antigens (24), including with a herpes virus type 2 glycoprotein (gD2) (25), ovalbumin (24), and with allergens (17). With the latter application, a formulation of dOMV_B_ containing mite allergens from *Dermatophagoides siboney* has been shown to be effective in a preclinical trial in controlling allergic reaction (26) and is currently undergoing a Phase I clinical trial (27). Another formulation, Protollin™(Glaxo Smith Kline, GSK) has been developed that combines *N. meningitidis* outer membrane proteins (OMP) and LPS from *Shigella flexneri*. This formulation has been used as an intranasal adjuvant (28) and Phase I and II clinical trials have established that these vesicles are safe and well-tolerated (29). Additionally, a clinical trial of *N. meningitidis* OMP mixed only with influenza antigens (Proteasome-based influenza vaccine, GSK) demonstrated that the intranasal formulation was immunogenic and well-tolerated (30).

Overall, OMV developed from *N. meningitidis* have been successfully licensed or are undergoing clinical trial. Table 1 shows a summary of the most successful dOMV evaluated against meningitis. Other uses are also being found for these OMV and the following sections highlight the advances in R&D of OMV derived from other bacteria that demonstrate the versatility of these structures.

## OMV Vaccines Against *Bordetella pertussis*

Whooping cough or pertussis is a highly contagious respiratory disease caused by *Bordetella pertussis*. Despite high vaccination coverage with whole cell or acellular vaccines, pertussis has re-emerged not only in children, but also in adults, which can be an additional source of infection for infants (31, 32). Among the reasons offered to explain this resurgence is the waning of vaccine-induced immunity and the presumed low vaccine efficacy of acellular vaccines, which support the introduction of new vaccine candidates to confer a protective long-lasting immunity (33).

Pertussis proteoliposomes or OMV (dOMV_BP_) have been constructed from inactivated whole cells of *B. pertussis* strain 165. Characterization studies have shown that these vesicles are composed of several immunogenic antigens including, pertussis toxin, fimbriae 3, and pertactin (34). Additionally, the dOMV_BP_ vaccine was highly protective against the WHO strain 18323 in intracerebral and intranasal challenge models (34). Similarly, a group of researchers in Argentina have obtained nOMV_BP_ using a detergent-free process with wild and mutant strains (35, 36); these candidate formulations were also protective when evaluated using an intranasal challenge model.

## OMV Vaccines Against *Enteric* Pathogens

**Figure 1:**
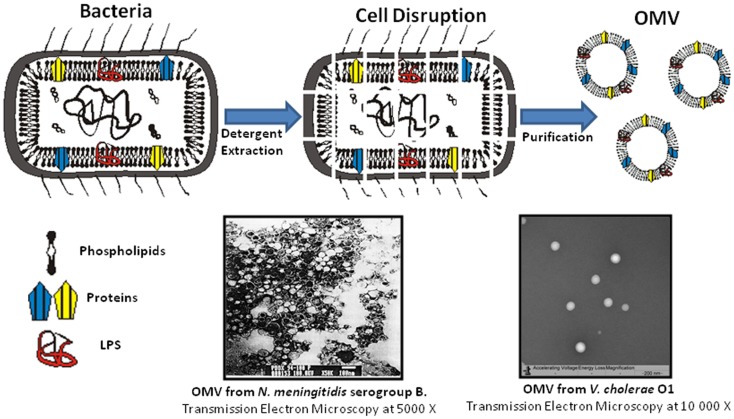
**Schematic representation of the dOMV extraction process and two micrographs of OMV obtained using this technology with two different detergents: OMV from *N. meningitidis* serogroup B extracted with deoxycholate (10) and OMV from *V. cholerae* O1 extracted with sodium dodecyl sulfate (37)**. OMV have a similar size and vesicle like structure. Differences observed between micrographs are mainly due to changes in magnification and stains used.

Enteric infections induced by pathogens are one of the main causes of death all over the world (6). The main bacterial agents are *Vibrio cholerae, Salmonella* spp., *Shigella* spp., and *Escherichia coli*. *V. cholerae* O1 proteoliposomes were the first vesicles (OMV_C_) obtained at the Finlay Institute from enteric pathogens. Figure 1 shows a micrograph of OMV_C_ from Perez et al. which demonstrated that these vesicles induced an antibody response with vibriocidal activity when administered via the nasal route (37). An OMV_C_ extraction process was developed with sodium dodecyl sulfate (SDS) detergent to achieve maximum recovery of LPS from the bacteria. Protein antigens with vaccine potential, such as OmpU and MSHA, were also found in OMV_C_ (37). In an alternative approach, Schild et al. obtained nOMV_C_ using a detergent-free method and demonstrated that intranasal and oral administration of these vesicles were immunogenic and protective in a model where the offspring of immunized female mice were infection challenged (38).

Selection of the detergent can be a critical step for extracting immunogenic OMV. LPS is the main antigen of enteric pathogens, but it is also a potent toxin with differing potency in Gram negative pathogens (39); therefore, detergent and purification steps, may differ according to the antigen that needs to be expressed or removed from the vesicles. Recently, the production of OMV obtained by detergent-free protocols has gained interest, because the generation of mutant strains, hyper expressing important protein antigens, and detoxified molecules may improve the yield, immunogenicity, and safety profile of the OMV (5, 16).

Several OMV extracts from different enteric pathogens have been evaluated at the Finlay Institute. A multivalent formulation that contained dOMV from *V. cholerae, S. enteritidis, S. typhimurium, Shigella sonnei*, and *S. flexneri* elicited high IgG (serum) and IgA (saliva) levels in mice and rats immunized orally (40). Additionally, OMV from enteropathogenic (EPEC) and enterotoxigenic (ETEC) strains of *E. coli* were evaluated in mice showing high specific antibody responses and heterologous cross-reactivity between them (41). Camacho et al. also demonstrated the potential of a mucosal candidate vaccine based on nOMV from *S. flexneri* (42).

Acevedo et al. demonstrated that a combination of dOMV_C_ with polysaccharide Vi (PsVi) from *S. typhi*, administrated via the nasal route, can induce immune responses at mucosal level, but also results in systemic specific IgG anti-PsVi responses as high as the parenteral PsVi vaccine vax-TyVi^®^ (Finlay Institute) (43). The potential use of combinations of different OMV or their capacity to be combined with antigens may have important impact in the future in the development of vaccines against enteric pathogens.

## OMV Vaccines Against Tuberculosis

Mycobacterial extracts have been widely used in vaccine developments, including the use of Freund’s complete adjuvant (FCA), which contains fragments from the mycobacteria. In particular, the mycobacterial cell wall contains a variety of antigenic and immunostimulatory molecules, such as peptidoglycan, arabinogalactan, mycolic acids, proteins, phosphatidylinositol mannosides, tiocerol, lipomanann, and lipoarabinomann, which activate dendritic cells via mannose and NOD2 receptors, among others (44, 45). All these components are important molecular effectors involved in the infection process and have been reported to induce protective responses in mice against TB (46, 47).

New formulations, which are safer than FCA, but still immunogenic, are also under evaluation. For example, RUTI consists of a *Mycobacterium tuberculosis* (MTB) protein extract and lipids, which are delivered in liposomes (48). Preclinical experiments show that RUTI is able to induce marked accumulation of antigen specific IFN-γ-producing CD4+ and CD8+ T cells, whereas BCG increases only the recruitment of CD4+ T cells. A short treatment regimen with chemotherapy (isoniazid) and RUTI is under evaluation in clinical trials. The reduction time in treatment as well as increased efficacy in chemotherapy will impact on the regression of the disease as well as the reduction of drug resistant MTB strains (48). Another, mycobacterial derivative with adjuvant and vaccine potential is the CAF01 formulation (49). The mycobacterium cord factor trehalose-6,6-dimycolate and its synthetic analog trehalose-6,6-dibehenate (TDB) are potent glycolipid immune stimulators that are recognized by a C-type lectin Mincle receptor (50). This signal activates dendritic cells, leading to cytokine production and up-regulation of co-stimulatory molecules (50). Incorporation of TDB in cationic liposomes (CAF01) together with the recombinant fusion protein Ag85B/ESAT-6 is a promising strategy against TB, developed by the Staten Serum Institute (Copenhagen, Denmark) (51).

dOMV derived from non-pathogenic mycobacteria have also been obtained. *M. smegmatis* and BCG have high levels of genomic and antigenic homology with MTB (52). Therefore, it is not surprising that proteoliposomes (dOMV) derived from both mycobacteria have induced cross-reactive immune responses against MTB antigens at cellular and humoral levels in mice (52, 53). Recent results have demonstrated that these candidates are as protective as BCG in challenge experiments conducted in mice (54).

## Conclusion

Outer membrane vesicles are very complex supramolecular structures. They contain immune stimulators (e.g., LPS, proteins, and DNA) and antigenic molecules that can be delivered to immune competent cells of the immune system to trigger maturation as well as activation signals. Therefore, OMV have an intrinsic adjuvant effect over loaded antigens from bacteria, but also over heterologous antigens that can be incorporated or combined in a single formulation. Altogether, the versatility to enable administration via the mucosal or parenteral route offers significant choice. The adjuvant potential and increased knowledge in the design of OMV over the last few decades will also enable the future development of the next generation of novel vaccine formulations.

## Conflict of Interest Statement

The authors declare that the research was conducted in the absence of any commercial or financial relationships that could be construed as a potential conflict of interest.
